# A novel method to assess heat transfer and impact of relevant physicochemical parameters for the scaling up of solid state fermentation systems

**DOI:** 10.1016/j.btre.2022.e00764

**Published:** 2022-09-19

**Authors:** Amélie Vauris, Sophie Valcauda, Florence Husson, Joëlle De Coninck

**Affiliations:** a Univ. Bourgogne Franche-Comté, L'institut Agro Dijon, PAM UMR A 02.102, 1 esplanade Erasme, Dijon F-21000, France; b Eurogerm, Parc d'activités bois Guillaume, 2 rue champ doré, Saint-Apollinaire F-21850, France

**Keywords:** Solid state fermentation, Heat transfer, Forced-aerated reactor, Physicochemical characterization, Carr index

## Abstract

Heat production during fermentation is undesirable. It can affect the growth of biomass, sporulation, products formation and the scaling-up. Physico-chemical properties of substrates influence heat and mass transfer in Solid State Fermentation. Heat is chemically produced into substrates without micro-organism to allow better reproducibility. A 2^4–1^ fractional factorial design is chosen to study the influence of four physicochemical parameters on heat transfer: Granulometry, Bulk Density, Carr Index (compressibility index) and Water Absorption Capacity. Results show that the two main physicochemical parameters which influence heat transfer are Granulometry and Carr Index. High Granulometry and low Carr Index have influence on maximum temperature reached during the test, warm-up speed and cooling speed. These two parameters allow efficient air flow through the substrate bed with large interparticle spaces enhancing exchange surface between air and particles. A substrate with these characteristics facilitates heat transfers in forced-aerated reactors.

## Highlights

• New method to study heat transfer in SSF under forced aeration.
• Granulometry and Carr index have the greatest impact on heat transfer.
• Substrates with low CI and high GR facilitate the scaling up of a SSF process.
• Bulk density has poor effectiveness in heat removal.

**Abbreviations**
BD: bulk density
CI: carr index
Cp: specific heat
DM: dry matter
GR: granulometry
HF: heat flow
MC: moisture content
SSF: solid state fermentation
WAC: water absorption capacity
WB: wheat bran

## 1 Introduction

Solid-state fermentation (SSF) can be briefly described as microbial fermentation which takes place in the absence or near absence of free water. This is a bioconversion process carried out on solid state substrate with moisture content about 30–80% [1], [2], [3]. It is being successfully employed to produce food enzymes such as lipases [4,5], xylanases [6], endoglucanases [6], amylases [7] or proteases [7, 8], protein isolation [9,10], and processing of animal feeds [11], [12], [13], [14]. Many secondary metabolites are also produced by SSF with applications in a wide variety of fields: pharmaceuticals, food, cosmetics, agriculture [15].

The microbial growth under aerobic conditions induces inconsiderable heat production that causes a fast increase of temperature [15,16]. A large quantity of metabolic heat is produced during SSF, up to 3200 kcal.kg^-1^ dry matter (DM) in composting systems [17] and a temperature gradient of 3 °C.cm^−1^ in tempeh fermentation [18,19]. Heat generation is directly related to the metabolic activities of the microorganisms, particularly respiration during growth, which is related to oxygen consumption and CO_2_ formation. In some case, temperature inside the bed can reach 70 °C [20]. Thus, the nutritional composition of the substrate is important in heat production.

This effect is undesirable especially in some biotechnological processes of heat sensible products or enzymes that can be heat-denatured. It also causes desiccation of substrate due to evaporation of moisture and affects the growth of the biomass, sporulation, and product formation.

In SSF, heat dissipation is hampered by the poor effective thermal conductivity of the porous media, normally made of organic materials [23]. The control of temperature under forced aeration is accomplished by adjusting aeration rate and temperature. If the temperature rises up during fermentation, increasing aeration rate and decreasing the temperature of the air promote cooling of the substrate [20].

In SSF, heat transfer is an important factor for the growth of the microorganism, product formation, and quality of the product [21]. For an efficient SSF scale-up it is necessary to have a deep knowledge of the process engineering: mass transfer phenomena and energy flow models, etc., and a wide experience on methodologies used to study physical properties affecting these phenomena [22].

Many types of reactors can run at laboratory-scale with small quantities of medium. But, the scale-up is complicated mainly by intense heat generation and heterogeneity in the system [24].

The desired final product yield and production speed in the SSF depends on the organism and substrate used. The physicochemical properties of substrate in SSF have been considered as the primary factors influencing the heat and mass transfer in SSF process, as well as fermentation performance [25,26]. Particle size is a critical factor in SSF. Large surface area contributes to accessibility of the solid matrix. Small particles contribute to larger surface area but will affect oxygen transfer. Large particles result in less accessibility but better for heat transfer [26]. Substrate density is also one of the key parameters [9,27]. In their modeling, Sangsurasak and Mitchell show that the bulk density can have an impact on heat transfer [28]. Dorta and Arcas [29] study the effect of packing density on spores yields of *Metarhizium anisopliae* cultivated in a mixture of rice bran and rice husk and observe that for low BD (from 0.270 to 0.357 g.cm^−3^), no significant differences take place on both the total biomass production and the spore yield. However, a significant reduction on the total accumulated biomass is observed when BD is increased up to 0.496 g.cm^−3^. These values are measured on dry substrates.

In SSF processes, evaporation makes very significant contributions to heat removal [28]. So Water Absorption Capacity (WAC) is considered important and must be taken into account. Kumar et al. [30] use *Aspergillus niger* to individually ferment Wheat Bran (WB), enriched by molasses or sucrose, and observe agglomeration in WB beds at moisture content (MC) as low as 65%. Poorna and Prema [31] study the production of endoxylanase from *Bacillus pumilus* cultivated in WB and found best result for 71.4% MC. According to the authors, low MC reduces swelling capacity of the substrate, increasing water surface tension and consequently reducing the water activity for the microbial metabolic requirement. In opposition, very high MC reduces interparticle spaces, leading to a decrease in the bed voidages and resulting in less space for microbial growth and deficient gaseous exchange. Substrate compaction becomes problematic when scaling up the process [24] and prevents heat removal.

The main objective of this study is to highlight the importance of the physicochemical characterization of substrates before starting a fermentation in solid medium and its impact on heat transfer during SSF process under forced aeration. Chemical heat is produced by adding calcium oxide in the moistened substrate for better reproducibility. An experimental design is applied to choose one or more methods of characterization the most relevant in heat transfer with forced-aeration reactors.

## 2 Materials and methods

### 2.1 Substrates

Coarse wheat bran, fine wheat bran, crushed corn, coarse ground corn, fine ground corn, cornmeal, crushed wheat, coarse ground wheat, fine ground wheat, buckwheat seeds, crushed buckwheat, precooked whole wheat, skinned whole wheat, precooked cracked wheat, roasted crushed wheat, roasted couscous, ground rice are provided by Eurogerm SA, Saint-Apollinaire, France.

### 2.2 Functional properties of substrates

#### 2.2.1 Granulometry (GR)

The granulometry is assessed by sieving, using different sieve mesh size (1000 µm, 800 µm, 500 µm and 250 µm). The content of each sieve is weighed and relative abundance is given as the percentage of total initial weight.

#### 2.2.2 Bulk density (BD)

Bulk density is determined according to the method described by Oladele A.K. and Aina, J.O. [32]. About 400 mL of sample is weighed and placed into a 1000 mL graduated measuring cylinder. The base of the cylinder is gently tapped until a constant volume is obtained. The bulk density (g.mL^−1^) is expressed as weight of substrate (g) per substrate volume (mL). BD is measured on substrates moistened at WAC value for each substrate.

#### 2.2.3 Carr index (CI)

Carr index (CI) or Carr's compressibility index is an indication of the compressibility of a powder [33]. A graduated cylinder of 250 mL volume is filled with about 200 mL of the granular material and weighed. The filled specimen is attached to the volumetric meter (J. Engelsmann, STAV II) and is subjected to a vertical tapping motion. The number of impacts is set at 2500 iterations. The bulk density of the granular material increases and tends towards maximum compactness. At the end of the test, the packed density of the granular material is obtained by dividing the weighed mass by the packed volume. Carr Index (CI) is based on bulk density (ρB) and tapped density (ρT) values: [CI = 100 × (ρT-ρB)/ρT]. CI is measured on substrates moistened at WAC value.

#### 2.2.4 Water absorption capacity (WAC)

Water absorption capacity is determined at room temperature based on a previously reported method [34]. About 100 g of sample is homogenized with excess water and let at room temperature during 1 h. The sample is then drained for 30 min. The results were expressed as a percentage of the retained water over total wet weight.

#### 2.2.5 Specific heat (Cp)

The specific heat, Cp, of the samples is determined by differential scanning calorimetry with the Mettler-Toledo DSC 1 calorimeter equipped with an FRS5 sensor and a Huber TC100 cooling system. A nitrogen flow of 50 mL.min^−1^ is used during the measurements. Approximately 20 mg of sample is analyzed in Tzero TA Instruments airtight crucibles. For each sample, three independent measurements are performed. The following temperature program is considered, with a temperature ramp of 10 °C.min^−1^: cooling from 25 °C to 10 °C; isothermal at 10 °C for 2 min; Heating from 10 °C to 90 °C. The specific heat of the samples is determined by the direct method, during the heating step: [C*p* = (*HF*sample−*HFblank)/m.β*] where HF sample is the heat flow of the sample, HF blank is the blank heat flow (empty sample crucible), m is the sample mass, and β is the heating rate of the sample. The specific heat of samples as a function of temperature gives the value of the specific heat of samples at 30 °C. To estimate the accuracy of the measurements, the specific heat of distilled water is also determined (4.26 ± 0.02 J.*g*^−1^.*K*^−1^ at 30 °C) and compared to the theoretical value (4.18 J.*g*^−1^.*K*^−1^), i.e. an error of about 2%. Cp is measured on substrates moistened at WAC value.

#### 2.2.6 Heat transfer

**Fig. 1:**
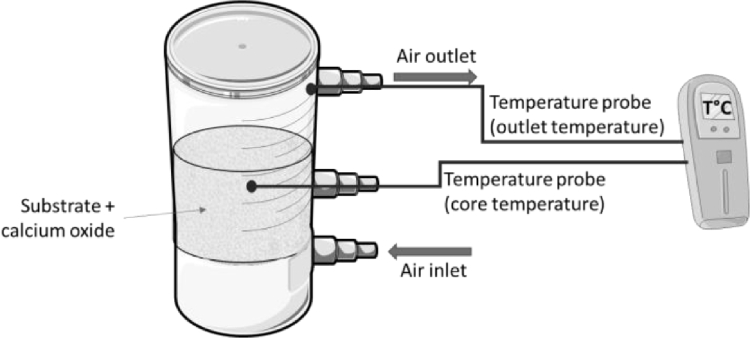
Schema of the mini-reactor of 1000 ml.

The SSF in the forced aeration condition is carried out using a 1000 mL mini-reactor (Fig. 1). The air flow-rate at 30 °C is adjusted to 3.0 ± 0.6 L.min^−1^. The thermos-anemometer (Kimo, LV111, France) is used to measure the velocity of outlet air from the mini-reactors respectively. One type T thermocouple is fixed to the wall of the outlet aeration tube and another type T thermocouple is placed inside the mini-reactor at the core of substrate to continuously measure temperature.

Each substrate has been soaked overnight at 4 °C to be at their water absorption capacity and then drained for 30 min. An amount depending on each substrate is placed in the mini-reactor to obtain a layer height of 6 or 10 cm. To generate heat and thus mimic metabolic heat, calcium oxide is added directly in the substrate in the proportion of ¼ (W/V) taking into account the water of the humidified substrate.

The temperature is recorded every 30 s until it decreases below 35 °C. On these records, maximum temperature, warm-up speed and cooling speed are determined. Maximum temperature is the highest value obtain during the test. Warm-up speed is the average rate of increase in temperature until reaching the maximum temperature and cooling speed is the average speed when temperature decreases between 45 °C and 35 °C.

## 3 Statistical analysis

### 3.1 Statistical treatment of data

The results of maximum temperature, warm-up speed and cooling speed are statistically performed using the software Minitab 19 (Minitab, LLC., USA).

One way analysis of variance (ANOVA) is applied after validating the feasibility of the test using variance analysis. Significance is established at *p* < 0.05 for significant results. ANOVA showing significant differences lead to the use of Tukey's multiple comparison test to group the sample.

A Pearson test is performed on GR, BD, CI, WAC and Cp to determine potential correlations between them. Significance is established at *p* < 0.05 for significant results.

### 3.2 Experimental design

Among physicochemical factors to characterize substrates, four are chosen to study heat transfer by a two-level factorial design [35]: GR, BD, CI and WAC.

These four factors are evaluated in eight experimental runs with a 2^4–1^ fractional factorial design. Factor levels are represented in Table 1, where the two levels of each variable are identified by either a minus (-) or a plus (+) sign. It is known that coarse and fine wheat bran have different behavior in SSF, especially in thick layer processes. GR level is chosen to differentiate these two levels. The wet BD is little used in the literature. According to our tests, densities vary between 400 and 1000 kg.*m*^−3^. The value 700 therefore represents a median value between these two extremes. CI is an indication of the capacity of a material to be compacted. Poor compaction indicates low cohesion between the substrate particles. The values of CI above 25% indicate important compressibility [36]. In SSF the moisture content varies from 30% to 80% and is often about 60% [1].

**Table 1:** Classes definition for the experimental design.

**Classes definition**				
**Parameter**	**-1 level**	**Value**	**+1 level**	**Value**
BD	low	<700 kg.*m*^−3^	strong	>700 kg.*m*^−3^
CI	Important compressibility	>25%	Low compressibility	≤25%
GR	fine	<50% (>800 µm)	coarse	>50% (>800 µm)
WAC	low	<60%	strong	>60%

BD: Bulk Density, CI: Carr Index, GR: Granulometry, WAC: Water Absorption Capacity.

Each trial is run with a mixture of these factors and is repeated.

This design has a resolution IV without aliases between main effects or between a principal effect and two factor combinations, but with aliases for combinations between two or more factors [37].

Obtained data are analysed by multiple regressing using Minitab 19 software (Minitab, LLC., USA). The variables with confidence levels lower than 5% are considered to significantly affect heat transfer.

## 4 Results and discussion

### 4.1 Heat transfer in a substrate model (wheat bran) to validate a new methodology

Very few recent publications studied heat transfers within an SSF system [38], [39], [40], [41]. These studies are based on modeling allowing more precise control of the equipment during process.

It is known that the particle size of the substrate can interfere with the fermentation process because the surface areas of the substrate particles is a limiting factor to fungal attack [42]. Substrates with smaller particles provide a higher surface area for the colonization of micro-organisms; however, extremely tiny particles can result in the agglomeration of the substrate, limiting the surface area of the substrate granule and affecting oxygenation and cultivation, thus leading to weak microbial growth. Otherwise, larger particles provide better aeration (oxygen diffusion) [42,43].

The decision is taken not to include this nutritional dimension which is well-known and specific to each microorganism and process. A new methodology is developed to mimic heat transfer without microorganisms using chemical self-heating and to validate this methodology, the wheat bran is tested with two different physicochemical characteristics (Table 2): coarse wheat bran and fine wheat bran. Three parameters are chosen to describe heat transfer: maximum temperature in the core of the substrate, rate of temperature rise until it reaches its maximum value and temperature drop rate.

**Table 2:** Maximum temperature, warm up speed and cooling speed for fine and coarse wheat bran at 6 cm and 10 cm layer height.

**Sample**	**T** °**C max (core) (** °**C)**	**Warm up speed (core) (** °**C.min^−1^)**	**Cooling speed (core) (** °**C.min^−1^)**
**Layer height: 6** **cm**			
Coarse wheat bran	61.03 ± 8.75^a^	3.07 ± 1.58^a^	0.54 ± 0.14^a^
Fine wheat bran	61.43 ± 2.43^a^	3.65 ± 0.64^a^	0.45 ± 0.08^a^
**Layer height: 10** **cm**			
Coarse wheat bran	64.70 ± 2.98^a^	3.64 ± 1.79^a^	0.57 ± 0.05^a^
Fine wheat bran	84.00 ± 2.97^b^	8.56 ± 1.05^b^	0.36 ± 0.04^a^

a,b: per column, values with different letters are significantly different (α = 0.05).

**Fig. 2:**
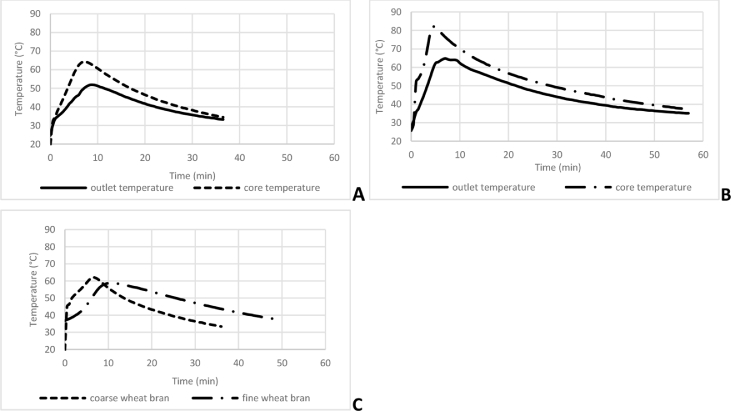
**A and B:** outlet and core temperatures measurements in the mini-reactor at layer height of 10 cm in coarse wheat bran (A) and fine wheat bran (B). **C:** core temperature measurements in the mini-reactor at layer height of 6 cm.

Results show that the self-heating method is validated, it allows to highlight the temperature rises and the elimination of calories (Fig. 2A and B). The temperature measured in the air outlet reflects the core temperature in forced aeration reactors. This makes it possible to adjust the fermentation parameters in order to avoid excessive temperature increases. Further results in this study are measured and calculated on core temperature.

Layer height has an important role in the elimination of calories (Fig. 2), confirming what is found in the literature. The larger the bed layer is, the greater the temperature rises [44]. Indeed, by comparing the values of maximum temperature, rate of rise and elimination of heat (Table 2), the values are much lower with a thin layer than a thick layer with forced aeration, regardless of the substrate. A higher height allows more area for heat dissipation but also a higher volume that implies more heat generation. A higher height also implies more weight and then more compaction in the reactor. The physicochemical composition of the substrate plays an important role in the removal of calories in forced-aerated thick-layer reactors [25]. Indeed, when two compositions of a substrate are compared, similar temperature values are noticed in thin layer. Maximum temperatures in core substrate rise 61.43 °C and 61.03 °C, warm-up speed are 3.65 °C.min^−1^ and 3.07 °C.min^−1^ and cooling speed are 0.45 °C.min^−1^ and 0.54 °C.min^−1^ for fine wheat bran and coarse wheat bran respectively. On the other hand, in a thick layer, great differences are noted. Core temperatures are higher with fine wheat bran (84.00 °C) as well as faster temperature rise (8.56 °C.min^−1^) and slower heat elimination (0.36 °C.min^−1^) compared to coarse wheat bran (64.70 °C, 3.64 °C.min^−1^ and 0.57 °C.min^−1^ respectively).

It is recognized that coarse wheat bran is a better substrate in SSF than fine wheat bran. It is therefore important to properly characterize the substrates before using them in SSF.

### 4.2 Physicochemical characterization of substrates

Twenty cereal substrates and mix are characterized (Table 3). To study the impact of physicochemical characterization, several factors are chosen. Except for the GR, all parameters are measured on substrates moistened at WAC value.

**Table 3:** Physicochemical characterization of the substrates and mix of substrates.

**substrate**	**GR(% >800** **µm)**	BD*(kg.*m*^−3^)	CI*(%)	**WAC (%)**	Cp*(J.*g*^−1^.*K*^−1^)
Coarse wheat bran	60.3	423.5 ± 5.6	33.5 ± 3.0	81.4 ± 1.0	3.6 ± 0.1
Fine wheat bran	32.1	493.5 ± 19.0	44.1 ± 8.5	76.1 ± 0.4	3.7 ± 0.2
Whole wheat precooked	100.0	726.9 ± 11.7	10.3 ± 1.1	30.3 ± 1.5	1.7 ± 0.1
Whole wheat skinned	100.0	717.7 ± 8.5	10.7 ± 1.3	31.2 ± 0.7	1.8 ± 0.1
Crushed wheat	99.6	753.6 ± 17.7	13.5 ± 2.9	47.3 ± 0.6	2.7 ± 0.1
Coarse ground wheat	46.0	1095.6 ± 15.3	1.9 ± 1.9	57.6 ± 0.4	3.3 ± 0.2
Fine ground wheat	8.0	980.7 ± 0.7	12.8 ± 0.7	62.5 ± 0.4	3.6 ± 0.1
Precooked craked wheat	84.5	737.2 ± 18.4	27.7 ± 2.8	53.9 ± 0.6	3.1 ± 0.1
Roasted ground wheat	65.6	796.4 ± 13.1	21.0 ± 1.2	57.1 ± 0.8	3.3 ± 0.2
Crushed corn	100.0	586.3 ± 9.5	5.8 ± 2.1	45.7 ± 0.6	2.4 ± 0.1
Coarse ground corn	80.8	718.6 ± 10.8	13.0 ± 2.9	55.5 ± 1.0	2.9 ± 0.1
Fine ground corn	57.0	687.5 ± 12.8	15.6 ± 2.8	62.2 ± 1.0	3.3 ± 0.2
Fine ground corn sieved	20.0	613.5 ± 10.8	17.0 ± 2.6	61.5 ± 0.9	3.4 ± 0.2
Cornmeal	1.3	901.9 ± 19.0	24.0 ± 9.3	58.8 ± 0.4	3.1 ± 0.2
Buckwheat seeds	100.0	775.4 ± 10.7	14.3 ± 2.8	50.2 ± 0.3	n.d.
Crushed buckwheat	69.9	959.2 ± 6.9	0.1 ± 0.1	60.3 ± 0.1	n.d.
Ground rice	5.0	848.6 ± 13.5	13.5 ± 1.8	54.1 ± 0.5	2.8 ± 0.1
Roasted couscous	99.2	714.7 ± 3.1	3.9 ± 2.2	74.7 ± 2.7	3.7 ± 0.2
80% ground rice + 20% coarse wheat bran	16.1	558.7 ± 7.6	44.1 ± 4.5	51.0 ± 0.2	3.2 ± 0.2
70% ground rice + 30% fine wheat bran	13.1	718.9 ± 17.1	34.36 ± 6.02	63.2 ± 0.9	3.2 ± 0.1

GR: Granulometry, BD: Bulk Density, CI: Carr Index, WAC: Water Absorption Capacity, Cp: Specific Heat.

n.d.: not determined.

⁎ BD, CI and Cp are measured on substrates moistened at WAC value.

First of all, moisture content is integrated into the study. The moisture content of the porous media is strongly related to the cultivation temperature, which is variable along the process due the heat metabolically generated. In this study, all the trials are realized at WAC. This is a characteristic of the substrate unless a chosen MC.

Small particles interfere with microbial respiration, leading to deficient microbial growth, while large particles limit the available surface for the microbial attack [45]. A compromise must be found to allow the best microbial growth as possible. Particle size is the second factor chosen.

Bulk density (BD), third factor chosen, affects SSF yields, especially due to microbial growth.

The bed depth of solid-state fermentation reactors is often limited by excessive compaction, which can reduce pore space and restrict permeability [46]. However, passage of air allows calories elimination during SSF with forced aeration. Air inlet is managed in temperature and moisture to maintain conditions in the substrate. When compaction is too important, air passes through preferential path and not through all bed. In this study, compaction is evaluated by Carr Index.

Cp is defined as the energy required to make the temperature vary of one unit of substrate through one degree. The Cp depends on moisture content and increase with the sample moisture [47]. This value is measured on the substrates of this study at their maximum water absorption capacity (Table 3). All the results depend essentially on the water content.

**Fig. 3:**
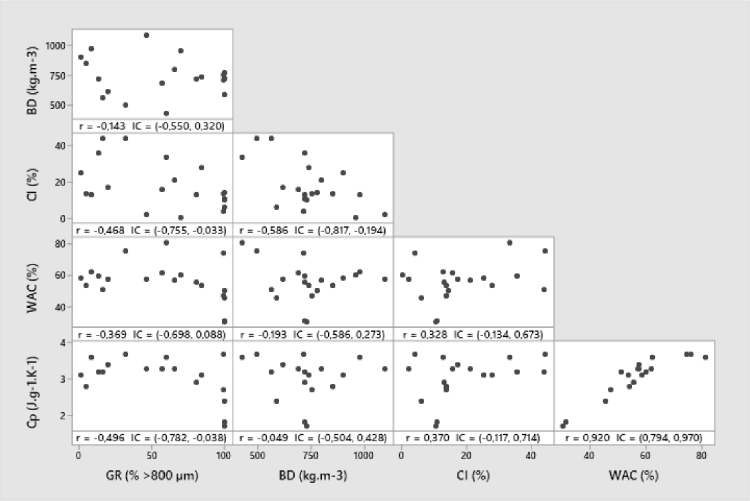
Pearson test on GR, BD, CI, WAC and Cp. GR: Granulometry, BD: Bulk Density, CI: Carr Index, WAC: Water Absorption Capacity. BD, CI and Cp are measured on substrates moistened at WAC value.

A Pearson test was performed on all these data (Fig. 3). The results show that wet Cp and WAC are very strongly correlated. The water content strongly influences the Cp, in accordance with the literature. Moreover, in the previous paragraph, the results show different behavior between fine wheat bran and coarse wheat bran. Indeed, the wet Cp of these two substrates are similar (3.6 J.*g*^−1^.*K*^−1^ for coarse wheat bran and 3.7 J.*g*^−1^.*K*^−1^ for fine wheat bran – Table 3). So the Cp of wet wheat bran does not play a role in heat transfer. The wet Cp is not retained for the characterization of the substrates and the water absorption capacity is preferred.

Granulometry, wet bulk density, wet Carr index and Water Absorption Capacity are finally selected in the study for their potential impact on air flow and heat removal. Impact and interactions of these characteristics on heat transfer are evaluated through an experimental design.

### 4.3 Experimental design to choose relevant methods to characterize substrates

To evaluate the impact of the characteristics of the substrates on heat transfer, a 2^4–1^ fractional factorial design is chosen. The software gives the characteristics of the substrates for the 8 trials. From these characteristics, the substrates or mix of substrates are determined (Table 4).

**Table 4:** Fractional factorial design.

**Trial n°**	**Substrates**	**Experimental design values**				**Real values**				**Maximum temperature**		Warm-up speed		Cooling speed
		**GR**	BD*	CI*	**WAC**	**GR (%)**	BD*(kg.*m*^−3^)	CI*(%)	**WAC (%)**	**(** °**C)**		**(** °**C.min^−1^)**		**(** °**C.min^−1^)**
Trial 1	70% ground rice + 30% fine wheat bran	-1	1	-1	1	13.1	718.9 ± 17.0	34.4 ± 6.0	63.2 ± 0.9	75.2		7.34		0.29
										73.3		6.50		0.32
Trial 2	80% ground rice + 20% coarse wheat bran	-1	-1	-1	-1	16.1	558.7 ± 7.6	44.1 ± 4.5	51.0 ± 0.2	75.2		5.31		0.29
										69.4		4.01		0.29
Trial 3	Precooked craked wheat	1	1	-1	-1	84.5	737.2 ± 18.4	27.7 ± 2.8	53.9 ± 0.6	63.7		4.19		0.47
										69.3		5.28		0.41
Trial 4	Coarse wheat bran	1	-1	-1	1	60.3	423.5 ± 5.6	33.5 ± 3.0	81.4 ± 1.0	62.0		14.94		0.56
										64.2		14.08		0.54
Trial 5	Crushed corn	1	-1	1	-1	100.0	586.3 ± 9.5	5.8 ± 2.1	45.7 ± 0.6	54.9		2.03		0.52
										56.5		1.85		0.49
Trial 6	Ground rice	-1	1	1	-1	5.0	848.6 ± 13.5	13.5 ± 1.8	54.1 ± 0.5	67.7		38.00		0.30
										66.1		38.00		0.31
Trial 7	Fine ground corn sieved	-1	-1	1	1	20.0	613.5 ± 10.8	17.0 ± 2.6	61.5 ± 0.9	76.2		33.48		0.30
										77.1		31.50		0.34
Trial 8	Roasted couscous	1	1	1	1	99.2	714.7 ± 3.1	3.9 ± 2.2	74.7 ± 2.7	60.0		5.36		0.66
										61.0		5.81		0.60

GR: Granulometry, BD: Bulk Density, CI: Carr Index, WAC: Water Absorption Capacity.

⁎ BD and CI are measured on substrates moistened at WAC value.

Maximum temperature, warm-up speed and cooling speed are measured for each trial and repeated to enhance the statistical resolution (Table 4). All these data are analyzed with the software Minitab 19 (Minitab, LLC., USA).

For each response, there are interactions between two or more characteristics with statistical significativity (Table 5). So, we need to study these interactions to analyze the results.

**Table 5:** Analysis of fractional factorial design.

	**Maximum temperature**		**Warm-up speed**		**Cooling speed**	
**Factor**	**Effect**	***P*-value**	**Effect**	***P*-value**	**Effect**	***P*-value**
GR	-11.1	0.000⁎⁎	-13.83	0.000⁎⁎	0.226	0.000⁎⁎
BD	0.1	0.931	0.41	0.293	0.004	0.783
CI	-4.1	0.006⁎⁎	11.80	0.000⁎⁎	0.044	0.011*
WAC	3.3	0.019*	2.54	0.000⁎⁎	0.066	0.001⁎⁎
GRxBD/CIxWAC	4.0	0.007⁎⁎	-3.48	0.000⁎⁎	0.004	0.783
GRxCI/BDxWAC	-2.6	0.049*	-17.66	0.000⁎⁎	0.029	0.061
GRxWAC/BDxCI	-2.6	0.051	4.17	0.000⁎⁎	0.051	0.005⁎⁎

GR: Granulometry, BD: Bulk Density, CI: Carr Index, WAC: Water Absorption Capacity.

BD and CI are measured on substrates moistened at WAC value.

⁎ Significant (*p*<0.05).

⁎⁎ Very significant (*p*<0.01).

**Fig. 4:**
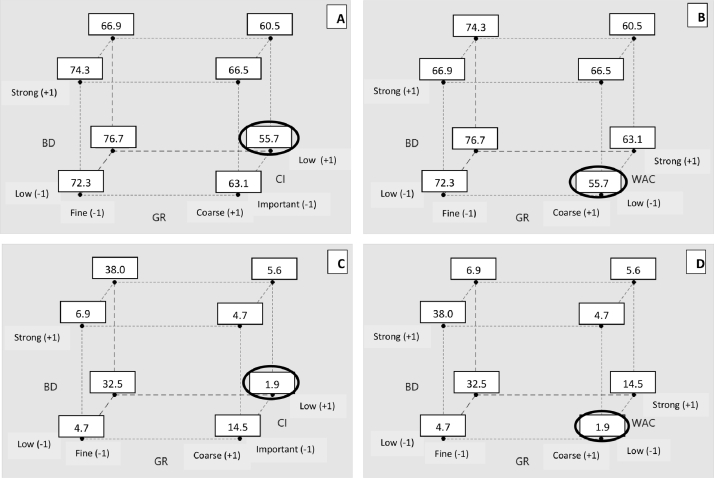
Geometrical representation of interactions for maximum temperature (A and B) and warm-up speed (C and D). GR: Granulometry, BD: Bulk Density, CI: Carr Index, WAC: Water Absorption Capacity BD and CI are measured on substrates moistened at WAC value. Peak values: average of tests (°C -A and B- or °C.min^-1^ -C and D-).

For the maximum temperature and warm up speed (Fig. 4), the value should be as low as possible, which indicates the most efficient heat removal. The lowest value for maximum temperature is 55.70 °C. The one for warm-up speed is 1.940 °C.min^−1^. These values are obtained for substrate with high GR, low CI and, with less effectiveness, low BD and low WAC.

**Fig. 5:**
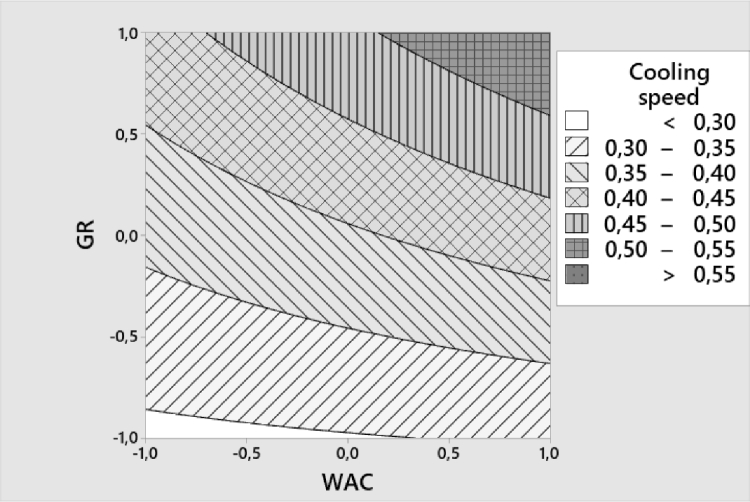
Contour curves for cooling speed ( °C.min^−1^) GR: Granulometry, WAC: Water Absorption Capacity.

For cooling speed, the value should be as high as possible to indicate the most efficient heat removal. Only one interaction has a statistical significativity for this parameter. The contour curves (Fig. 5) indicates that the most efficient heat removal is obtained with values greater than 0.55 °C.min^−1^ with a high GR, and, with less effectiveness, high WAC. Low CI have also a great impact on this parameter.

For these three results (maximum temperature, warm-up speed and cooling speed), high GR and low CI are the two main parameters that allow better heat transfer. These two parameters allow efficient air flow through the substrate bed with large interparticle spaces enhancing exchange surface between air and particles. Some authors show that better fermentations are obtain with high granulometry [30,31,48,49]. The particle size determined in each of these studies allows the best mass and heat transfer while allowing access to nutrients for microorganisms.

Without evaluating its value, Kumar et al. [30] observed the impact of compressibility on fermentation. Indeed, this author observes that for two substrates with equivalent humidity, wheat bran exhibits an agglomeration of particles unlike sugarcane bagasse. This phenomenon has an impact on the growth of the microorganism, heat and mass transfer and the production of metabolites.

In this study, the importance of compressibility in SSF is highlighted. This characteristic is important in scaling up and show the capacity of the substrate to maintain its structure in thick-layer reactors. Carr index can evaluate compressibility of substrates and gives information on heat removal during a SSF process. The capacity of a substrate to compress results in the reduction of interparticle spaces, thus limiting the exchanges between air and particles. The heat produced during fermentation can then no longer be evacuated, leading to an increase in temperature during cultivation.

For maximum temperature and warm up speed, low BD and low WAC have less effectiveness on heat removal. For cooling speed, only high WAC has less effectiveness. Evaporation is one of the phenomenon that allow heat removal in SSF under forced aeration [50].

Among the cereal substrates analyzed, which have the best-defined criteria, i.e. high particle size and low compressibility, nine substrates have some potential for the SSF process (Table 3): whole wheat precooked, whole wheat skinned, crushed wheat, roasted ground wheat, crushed corn, coarse ground corn, buckwheat seeds, crushed buckwheat and roasted couscous. These substrates will facilitate the scaling-up of the SSF process, in particular in thick-layer reactors. The coarse wheat bran which has been tested is not retained for its compressibility higher than the standard adopted because it could cause significant compaction in thick-layer reactors. If particle size is still studied in SSF process, compressibility index is a new parameter which could easily be measured. It requires only a volumenometer which is a current tool in cereal or pharmaceutical industries.

## 5 Conclusion

All the results underline the importance of the physicochemical characterization of the substrates for fermentation in a solid medium in a forced-aeration reactor. The characteristics measured on substrates can give information on their capacity to dissipate heat during a SSF process. In this study, a method with addition of calcium chloride to mimic heat production is used. This method allows the study of heat removal without micro-organism whose heat production depends on the development of the strain. The particle size and Carr index determinations show the greatest impacts on heat removal. These two parameters allow efficient air flow through the substrate bed with large interparticle spaces enhancing exchange surface between air and particles. While particle size has already proven its importance in the literature, the Carr index has been little emphasized and the results of this work prove that it must be considered like particle size. In forced-aerated reactors, heat transfer will be facilitated by using a substrate having a large particle size and a low Carr index.

## Funding

This work was supported by Eurogerm S.A.S.

## Declaration of Competing Interest

None.

## Data Availability

Data will be made available on request.
